# Supplementary material for: Decellularized Skin Extracellular Matrix (dsECM) Improves the Physical and Biological Properties of Fibrinogen Hydrogel for Skin Bioprinting Applications
Source: Nanomaterials (Basel). 2020 Jul 29;10(8):1484. doi: 10.3390/nano10081484 (PMC7466410; doi:10.3390/nano10081484)
Supplement: Supplementary file 1 [file nanomaterials-10-01484-s001.pdf]

Article

Decellularized Skin Extracellular Matrix (dsECM) Improves the Physical and Biological Properties of Fibrinogen Hydrogel for Skin Bioprinting Applications

Adam M Jorgensen, Zishuai Chou, Gregory Gillispie, Sang Jin Lee, James J Yoo, Shay Soker, Anthony Atala

Supplementary Materials:

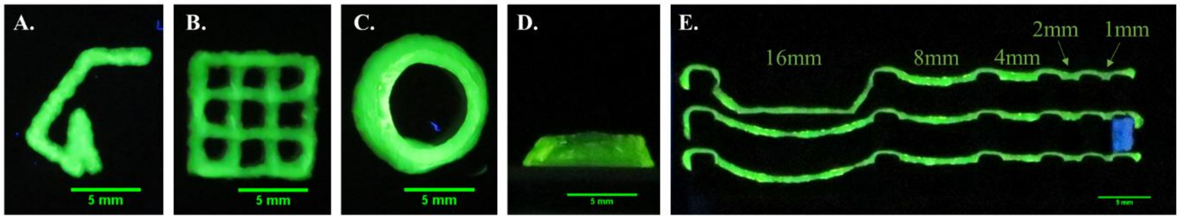

F.	Artifact Test	Fibrinogen	Fibrinogen + 1% dsECM	Fibrinogen + 2% dsECM	*p*-value
A.	Turn Angle (125°)	125.4 ± 2.6	126.4 ± 0.5	129.5 ± 2.0	*p* = 0.617
	Turn Angle (90°)	88.3 ± 0.2	87.7 ± 4.0	84.2 ± 3.5	*p* = 0.617
	Turn Angle (55°)	59.1 ± 1.3	56.7 ± 2.0	59.7 ± 1.1	*p* = 0.617
	Turn Angle (20°)	22.9 ± 2.8	20.2 ± 3.2	19.5 ± 1.8	*p* = 0.617
	Filament Thickness	1.2 ± 0.1	1.1 ± 0.1	1.1 ± 0.1	*p* = 0.152 *
	Filament Uniformity	1.0 ± 0.0	1.0 ± 0.0	1.0 ± 0.0	*p* = 0.317
B.	Crosshatch Pore Size	1.6 ± 0.3	2.6 ± 0.2	2.2 ± 0.1	*p* = 0.002 ***
	Crosshatch PR Value	0.94 ± 0.01	0.92 ± 0.01	0.94 ±0.01	*p* = 0.052 *
C.	Tube Wall Thickness	2.2 ± 0.3	1.8 ± 0.2	2.1 ± 0.2	*p* = 0.126 *
	Tube Radial Accuracy	0.9 ± 0.0	0.9 ± 0.0	0.9 ± 0.0	*p* = 0.592
	Average Tube Height	2.6 ± 0.1	2.2 ± 0.1	2.4 ± 0.0	*p* = 0.003 ***
	Average Tube Width	9.7 ± 0.5	9.3 ± 0.1	9.1 ± 0.0	*p* = 0.076 *
E.	Deflection Overhang (16mm)	-3.0 ± 0.0	-2.5 ± 0.4	-2.8 ± 0.4	*p* = 0.007 ***
	Deflection Overhang (8mm)	-0.9 ± 0.1	-0.8 ± 0.2	-0.9 ± 0.1	*p* = 0.832
	Deflection Overhang (4mm)	-0.5 ± 0.1	-0.4 ± 0.1	-0.4 ± 0.0	*p* = 0.623
	Deflection Overhang (2mm)	-0.3 ± 0.0	-0.3 ± 0.0	-0.2 ± 0.0	*p* = 0.940
	Deflection Overhang (1mm)	-0.2 ± 0.0	-0.2 ± 0.0	-0.1 ± 0,0	*p* = 0.987

Figure S1: Artifact printability data. (**A**) Turn accuracy, (**B**) crosshatch, (**C**) tube width, (**D**) tube height, and (**E**) overhang deflection tests were performed. (**F**) A table with detailed results of each printability test by hydrogel type. Data are presented as the mean ± the SD, with the associated p-value.

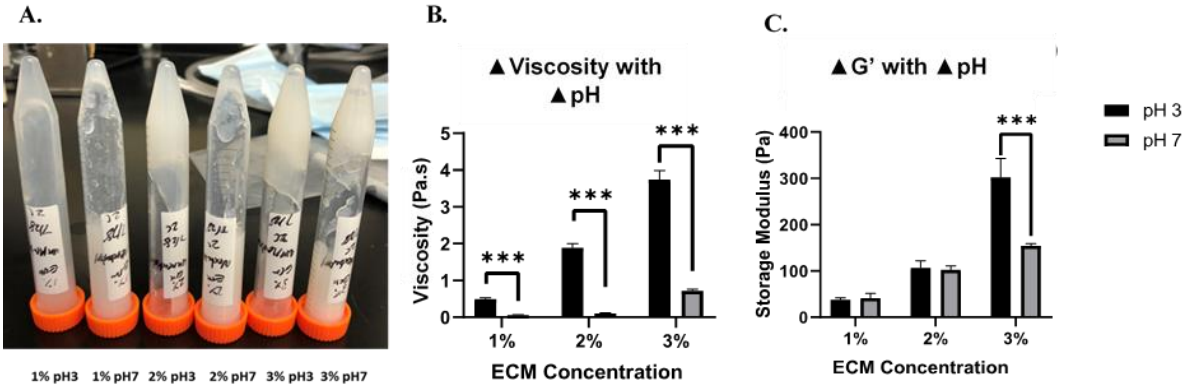

Figure S2: pH effect on human skin ECM rheological properties. (**A**) Image of various ECM solutions at pH3 and pH7. Phase separation was observed in ECM solutions at pH7, (**B**) average viscosity, and (**C**) average G′ of various ECM solutions at pH3 and pH7.
